# Oral Administration of *Prunella vulgaris L* Improves the Effect of Taxane on Preventing the Progression of Breast Cancer and Reduces Its Side Effects

**DOI:** 10.3389/fphar.2018.00806

**Published:** 2018-08-03

**Authors:** Jixue Zhao, Degang Ji, Xujie Zhai, Lirong Zhang, Xiao Luo, Xin Fu

**Affiliations:** ^1^Department of Pediatric Surgery, The First Hospital of Jilin University, Changchun, China; ^2^Department of Hepatobiliary Pancreatic Surgery, China-Japan Union Hospital of Jilin University, Changchun, China; ^3^Department of Breast Surgery, China-Japan Union Hospital of Jilin University, Changchun, China; ^4^Department of Pathology, China-Japan Union Hospital of Jilin University, Changchun, China; ^5^Department of Nursing, China-Japan Union Hospital of Jilin University, Changchun, China

**Keywords:** PVL, taxanes, pathologic complete response, disease-free survival, breast cancer, side effects

## Abstract

We aimed to explore the efficacy and safety of *Prunella vulgaris L* (PVL) combined with taxane for treatment of patients with breast cancer (BC). The main ingredients of PVL were analyzed by high-performance liquid chromatography (HPLC). In the experiment, 424 patients with BC were evenly assigned into two groups: experimental group (EG, oral administration of PVL and taxane) and control group (CG, oral administration of placebo and taxane). The primary endpoint was pathologic complete response (pCR), which was evaluated using Miller and Payne system. The secondary endpoints included adverse events (AE) and overall survival (OS), which were evaluated by Common Terminology Criteria for Adverse Event version and Kaplan–Meier curves, respectively. Response Evaluation Criteria in Solid Tumors was used to evaluate the clinical efficacy of PVL. Estrogen receptor (ER) status was also measured. The main side effects were compared between the two groups. The main ingredients of PVL were caffeic acid and rosmarinic acid, which both exert anti-tumor properties. The average follow-up time was 41 months. Eighteen and 31 patients dropped out from EG and CG, respectively. Overall, pCRs were detected in 94 cases (25.1%), comprising 61 cases (31.4%) from EG and 33 cases (18.2%) from CG (*P* < 0.05). PVL treatment improved the pCR rate and OS time compared with those in CG (*P* < 0.05). The 3-year OS rates were 86.5 and 77.2% in patients from EG and CG, respectively (*P* < 0.05). Moreover, ER status was associated with pCR rate and could be an independent prognostic factor in BC. Moreover, treatment with PVL prevented side effects, namely, neutrophil-reduced fever and anemia caused by chemotherapy. Hence, chemotherapy using PVL and taxane could be a safe and effective treatment for patients with BC. PVL may be a potential adjuvant medicine for BC treatment.

## Introduction

Breast cancer (BC) has become the most common malignancy in women. About 63,960 cases of carcinoma in female breast and 87,290 cases of melanoma were diagnosed in 2018 (Siegel et al., 2018). Although the prognosis of BC is simple and more accurate than other malignant tumors, its treatment will become extremely complicated once it recurs and metastasizes. The best option for controlling BC is early diagnosis and effective treatment with few side effects. In this regard, standardization of early BC treatment is important. Early treatment can considerably reduce the risk of recurrence and metastasis of BC and thus increase patient’s survival rate and time.

The principles for treatment of BC at different stages have been continuously refined. The commonly used methods are chemotherapy (Wang et al., 2018), radiotherapy (Al-Hammadi et al., 2018), surgery (Kim et al., 2018), targeted therapy (Jing et al., 2018), endocrine therapy (Anderson, 2018), and combination therapy (Ha et al., 2018). BC should be effectively controlled as soon as possible to minimize or delay its recurrence and metastasis. The National Comprehensive Cancer Network (NCCN) guidelines recommend neoadjuvant therapy for early and locally advanced BCs (Reyngold et al., 2014). This therapy aims to comprehensively control BC. Neoadjuvant therapy for BC was first used in patients with inoperable locally advanced BC (Goble and Bear, 2003). Given the technological development of comprehensive BC treatment in recent years, neoadjuvant therapy has been gradually applied to patients with early stage operable BC (Gollamudi et al., 2016). Although neoadjuvant treatment started relatively late, it has significant advantages over adjuvant treatment. First, neoadjuvant therapy can reduce the tumor burden and leads to successful subsequent local surgical treatment. This protocol may also reduce local planting and other dissemination of BC that may occur during the surgery. Second, the efficacy of neoadjuvant therapy can provide guidance for follow-up systemic therapy. Preoperative therapy can also eliminate whole body micrometastases and reduce their postoperative occurrence (Watanabe et al., 2017; Silva et al., 2018). Although neoadjuvant treatment for BC has been widely reported, this malignancy remains difficult to control (Nakayama et al., 2018; Patel et al., 2018). Hence, new medicine for neoadjuvant treatment must be explored.

*Prunella vulgaris L* (PVL) mainly contains flavonoids (Fazal et al., 2016; Qu et al., 2017), pentacyclic triterpenes (Yu et al., 2015), organic acids (Psotova et al., 2003), polysaccharides (Du et al., 2016), and volatile oils (Zhai et al., 2014). PVL extract exert a variety of biological activities, including regulation of tumor metastatic microenvironment (Su et al., 2016), antioxidant effect (Fazal et al., 2016), and anti-inflammatory effect (Hwang et al., 2013). Oral administration of PVL can effectively reduce the symptoms of gingivitis (Adamkova et al., 2004). PVL can also suppress the migration and invasion of human liver cancer cells (Kim et al., 2012). Ethyl acetate extracts of PVL downregulate the protein expression levels of B-cell lymphoma protein-2 (Bcl-2) and pro-angiogenic vascular endothelial growth factor (VEGF) and are thus potent anticancer agents for treatment of gastric cancer (Tan et al., 2015). PVL extracts also exhibit anti-estrogenic properties and can be used as therapeutic agents for treatment of estrogen-dependent tumors (Kim et al., 2014). PVL polysaccharide can inhibit human breast carcinoma-associated fibroblasts (CAFs) by inhibiting the expression of basic fibroblast growth factor (bFGF) and the growth of BC cells (Hao et al., 2016).

Docetaxel and taxane are commonly used for chemotherapy of BC and have been explored for combination therapy (Cocconi et al., 2018). PVL may improve the therapeutic effects of combination therapy. The current neoadjuvant chemotherapy program is based on the combination of PVL and taxane-based drugs. The present study aimed to assess the efficacy and safety of PVL combined with taxane for treatment of patients with BC.

## Materials and Methods

### High-Performance Liquid Chromatography (HPLC) Analysis of PVL

*Prunella vulgaris L* oral solution was purchased from Shandong Xianhe Pharmaceutical Co., Ltd. (Dongying, China). Standard solutions of caffeic and rosmarinic acids were obtained from sigma. The ingredients of PVL were analyzed using Shimadzu LC-2010A HPLC system with a size exclusion chromatography column (Shodex SB-804 HQ, Showa Denko, Kawasaki, Japan). The following chromatographic conditions were used: Elite Hypersil 18 column (5 pm, 250 nm × 4.6 nm); mobile phase: methanol−3.1% formic acid (18:82, 0−20 min) and methanol−0.1% formic acid (32:68, 20−60 min); flow rate, 1.0 ml/min; column temperature, 30°C; detection wavelength, 330 nm; and number of theoretical plates of caffeic acid and rosmarinic acid ≥5,000.

### Participants

All procedures were adhered to the Declaration of Helsinki and approved by the Human Research Ethical Committee of China-Japan Union Hospital of Jilin University (Changchun, China; Approval No. 2015CCX012). All patients with early and locally advanced BC were selected from June 2015 to October 2016 and would receive neoadjuvant chemotherapy at the Breast Cancer Center of our hospital. All patients were informed of the experimental procedures and signed a consent form for chemotherapy. All biopsies were extracted by using an automatic biopsy instrument (Manan Medical Products, Wheeling, IL, United States) with biopsy needles. Ultra-sound was used as an imaging guide method. The lesion was localized by ultrasound and incised at the puncture site by penetrating a biopsy needle. The biopsy material was immediately fixed in formalin for 2 days. The tissues were microscopically classified according to invasive ductal, lobular, mix, and others (El Sharouni et al., 2017).

### Inclusion Criteria

All patients were aged from 20 to 75 years old and only female were considered here. Tumor stage was from T1 to T4. Hormone receptor (HR) and or human epidermal growth factor receptor 2 (HER2) could be detected. Positive MDR-1 Pgp expression could be detected in BC patients. Patients with BC had definitive status of estrogen receptor (ER), progesterone receptor (PgR), and human epidermal growth factor receptor 2 (HER2). ER and PgR were detected by using Human ER Alpha ELISA Kit (Cat. No. LS-F4409-1) and Human PgR ELISA Kit (Cat. No. EKU06800) from Biocompare (South San Francisco, CA, United States). The HER2^+^ subtype was confirmed through fluorescence *in situ* hybridization test. The defined negative values for ER and PgR were <10% of the cells with positive nuclear staining for HER2. Patients were considered suitable for neoadjuvant therapy if they satisfy the following criteria: Invasive BC was diagnosed histologically in all patients by core biopsy. All subjects had detectable breast tumors with least 5 cm, positive lymph node status, in the trials by palpation, ultrasound, or mammography. The patients were willing to receive neoadjuvant treatment of oral administration of PVL and taxane chemotherapy. The patients underwent complete neoadjuvant treatment, and their clinical data were obtained. Cardiac function was measured by electrocardiograms (ECG). A person’s circulatory system was measured by arterial pulses and blood pressure. Liver function was measured using indocyanine green (ICG) clearance. Kidney function was measured by estimated glomerular filtration rate (eGFR). Prior to and after the therapy, circulating system (<50 mm Hg) (Thorin-Trescases et al., 2018) and liver (ICG > 10.8% per minute) (De Gasperi et al., 2016) and kidney function (eGFR ≥ 90 ml per minute per 1.73 m^2^) (Hayek et al., 2015) were normal. ECG showed normal heart function.

### Patient Grouping

Docetaxel and taxane were purchased from Cisen Pharmaceutical Co., Ltd. (Jining, Shandong). All 424 patients were assigned into two groups: experimental group [EG, patients received PVL (10 ml/time, twice daily), docetaxel (75 mg/m^2^, dl/21d), and taxane (175 mg/m^2^, dl/21d)] and control group [CG, patients received placebo, docetaxel (75 mg/m^2^, dl/21d), and taxane (175 mg/m^2^, dl/21d)].

All patients received blood routine examination 5 days after chemotherapy. The numbers of white blood cells and absolute neutrophils were determined by the clinician to assess if Neulasta (Amgen, CA, United States) will be used. Patients received injection of pegylated recombinant human granulocytes each week. The next cycle lasted to a maximum of 35 days after the last chemotherapy. Chemotherapy started when the blood system, liver function, and kidney functions were normal. All patients received at least four cycles of neoadjuvant chemotherapy. The surgery was performed 4 weeks after the end of the last chemotherapy session. Patients tested positive for estrogen and progesterone were subjected to endocrine therapy after surgery. Follow-up data of the patients were obtained by telephone call or from outpatient or inpatient medical record systems. The last follow-up was October 28, 2016.

### Primary Endpoint

The primary endpoint was defined as the time from the surgery to the time of recurrence or metastasis of BC. The endpoint was mainly confirmed by the hospital system or patient’s family members. Response evaluation criteria in solid tumors (RECIST) was used to evaluate the clinical efficacy, and the Miller and Payne system were used to evaluate the pathologic complete response (pCR).

### Secondary Endpoint

The secondary endpoint included adverse events (AE) and overall survival (OS), which were evaluated by Common Terminology Criteria for Adverse Events version (CTCAE) and Kaplan–Meier curves, respectively. Clinical efficacy was assessed by RECIST.

### Evaluation of Therapeutic Outcomes

The therapeutic effects were assessed according to the prognostic relevance of pCR. ER and PgR were regarded as positive if more than ten percent cells were stained positive. HER2 status was measured by using immunohistochemistry. BC subtypes were measured based on St. Gallen panelists (Jackisch et al., 2015). Therapeutic outcomes were evaluated using RECIST (**Tables 1, 2**) every 6 weeks. Miller and Payne system was used to evaluate the pathology of neoadjuvant therapy (**Table 3**). The toxicity of the therapy was assessed according to the National Cancer Institute Common Toxicity Response Scale (NCI-CTC) (Denis et al., 2003). The main parameters are as follows: pCR rate, which was defined as the proportion of patients without invasive cancer tissues in the breast and axillary lymph node specimens after surgical resection; survival rate, which was assessed by Mantel-Haenszel method; and disease-free survival (DFS), which was defined as the time from surgery to recurrence or metastasis of BC.

**Table 1:** Evaluation criteria of therapeutic efficacy of solid tumor.

Terms	Definition
**Target lesion evaluation**	
Complete remission, CR	Target lesion disappeared
Partial remission, PR	Reduction of axial length of lesion baseline >30%
Stable disease, SD	Reduction of axial length of lesion baseline <30% or increase of axial length of lesion baseline >20%
Progression disease, PD	Progression disease
**No target lesion evaluation**	
CR	All non-target lesions disappeared and tumor markers were at normal levels
SD	One or more non-target lesions and/or tumor markers higher than normal levels
PD	One or more new lesions or/and non-target lesions were present

**Table 2:** Evaluation criteria of overall efficacy.

Target lesions	No-target lesions	New lesions	Overall efficacy
CR	CR	No	CR
CR	No CR/No PD	No	PR
CR	NE	No	PR
PR	No PD or NE	No	PR
SD	No PD or NE	No	SD
PD	any	Yes or No	PD
Any	PD	Yes or No	PD
Any	any	Yes	PD

**Table 3:** Grading system of pathological response Miller and Payne.

Grades	Classification definition
G1	Infiltrating cancer cells did not change or only individual cancer cells changed, but the total number of cancer cells did not decrease
G2	Infiltrated cancer cells decreased slightly, but the total number was still high, and cancer cells decreased by no more than 30%
G3	Infiltrating cancer cells reduced by 30 to 90%
G4	Infiltrating cancer cells significantly reduced by more than 90%, leaving only scattered small-cancerous cells or individual cancer cells
G5	Tumor tumors have no infiltrating cancer cells, but there may be ductal carcinoma *in situ*

### Statistical Analysis

The post-hoc power percentage (94.25%) showed that the power of sample size was adequate. Categorical data and the proportion of patients were compared by Fisher’s exact test between two groups. Chi-square test was used for counting number and Student’s *t*-test for other data. Univariate and multivariate logistic regression analyses were conducted to determine the factors associated with pCR. Kaplan–Meier curves were used to evaluate OS. All statistical analyses were conducted using SPSS software (version 20.0, SPSS Inc., IBM, NY, United States).

## Results

### Main Ingredients of PVL Oral Solution

**FIGURE 1:**
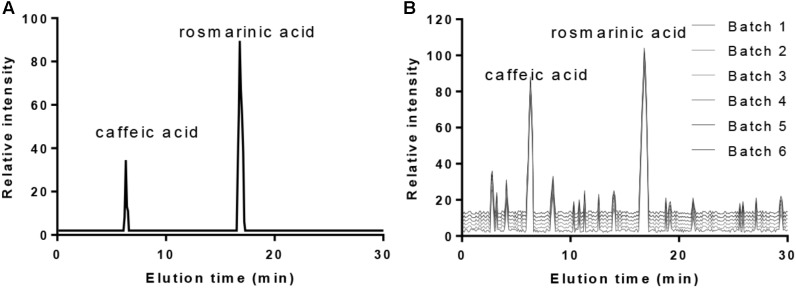
The main ingredients of PVL oral solution. **(A)** The standards of caffeic acid and rosmarinic acid. **(B)** The main ingredients of PVL from six different batches.

High-performance liquid chromatography analysis with the standards (**Figure 1A**) showed that the main ingredients of PVL were caffeic acid and rosmarinic acid (**Figure 1B**). The ingredients were stable from different batches.

### Clinical Characteristics

A total of 424 patients with BC consecutively confirmed by pathological analysis were enrolled in the study. All of the patients received treatment with or without PVL. The median age was 48.3 (23.1−75.1) years. All patients were followed up, and the median follow-up time was 41 months. Forty-nine patients were lost in the follow-up. Surgical treatment was performed 4 weeks after the chemotherapy. Fifty-one cases (26.6%) underwent breast conservation, and 141 cases (73.4%) underwent breast removal. Before the treatment, the statistical differences for the types of histology were insignificant between two groups (**Table 4**, *P* > 0.05). All clinical parameters were not significantly different between EG and CG (**Table 4**, *P* > 0.05).

**Table 4:** Clinical characteristics of breast cancer patients between two groups.

	EG, *n* = 212	CG, *n* = 212	*χ*^2^ and *t* value	*P*-values
Age, year	47.7 ± 24.6	49.1 ± 26.0	0.345	0.621
Before menopause	125(59)	128(60.4)	0.088	0.766
Type of histology				
Invasive ductal	187(154.5)	169(139.7)	5.705	0.127
Invasive lobular	14(11.6)	25(20.7)		
Mixed	6(5)	10(8.3)		
Others	5(4.1)	8(6.6)		
**T staging, *n* (%)**		
T1 (= 2 cm)	53(25)	49(23.1)	0.232	0.972
T2 (2 cm < T ≤ 5 cm)	124(58.5)	126(59.4)		
T3 (> 5 cm)	33(15.6)	35(16.5)		
T4 (Chest wall or skin infiltration)	2(0.9)	2(0.9)		
**N staging, *n* (%)**		
N0	56(26.4)	58(27.4)	0.341	0.952
N1	117(55.2)	113(53.3)		
N2	20(9.4)	23(10.8)		
N3	19(9)	18(8.5)		
ER situation, negative, *n* (%)	60(28.3)	58(27.4)	0.047	0.828
PR situation, negative, *n* (%)	75(35.4)	79(37.3)	0.163	0.686
HER2 situation, negative, *n* (%)	158(74.5)	151(71.2)	0.585	0.444
**Breast surgery**		
Conserving, *n* (%)	53(25)	48(22.6)	0.325	0.569
Removal, *n* (%)	159(75)	164(77.4)		

### Clinical Therapeutic Evaluation of BC Therapy

A total of 375 patients with BC completed the experiment. The total effective rate of all the patients was 81.6% (306/375). The statistical differences for CR, PR, SD, and PD were significantly different between the two groups (*P* > 0.05, **Table 5**). Overall, pCRs were detected in 94 cases (25.1%), comprising 61 cases (31.4%) from EG and 33 cases (18.2%) from CG (*P* < 0.05, **Table 5**). PVL improved the pCR of patients with BC, suggesting that combination treatment with PVL will be better than chemotherapy with taxane alone.

**Table 5:** Therapeutic evaluation of breast cancer.

Parameters	EG, *n* = 194	CG, *n* = 181	*χ*^2^ values	*P*-values
CR, *n* (%)	18(9.3)	6(3.3)	5.431	0.020^∗^
PR, *n* (%)	125(64.4)	78(43.1)	17.173	0^∗^
SD, *n* (%)	31(16)	45(24.9)	4.572	0.033^∗^
PD, *n* (%)	3(1.5)	14(7.7)	8.286	0.004^∗^
pCR, *n* (%)	61(31.4)	33(18.2)	9.223	0.002^∗^
HR^+^/HER2^−^	15(7.7)	11(6.1)	0.397	0.528
HR^−^/HER2^+^	8(4.1)	5(2.8)	0.519	0.471
HR^+^/HER2^+^	7(3.6)	4(2.2)	0.643	0.423
HR^−^/HER2^−^	21(10.8)	12(6.6)	2.053	0.152

*^∗^P < 0.05 vs. the CG group.*

### Exclusion Criteria

The following patients were excluded from the study: pregnant and lactating women; those with distant metastasis; those who underwent focal resection or local radiotherapy before treatment; those with malignant tumors, including advanced BC, inert non-melanoma skin cancer, and cervical cancer *in situ*; and those with serious systemic diseases.

### Factors Affecting pCR Rates

The following main factors were considered: age, menstrual status, ER, PR, HER2 status, T staging, and N staging. Univariate analysis showed that ER-negative patients had higher pCR rates than ER-positive patients (41.2 vs. 12.3%, *P* < 0.05). PR-negative patients had higher pCR rates than PR-positive patients (34.6 vs. 12.4%, *P* < 0.05). Age, menstrual status, T staging, and N staging were significantly associated with pCR rates (**Table 6**, *P* < 0.05). Multivariate analysis showed that ER status was also associated with pCR rate. T staging and N staging were significantly correlated with pCR rate (**Table 7**, *P* < 0.05).

**Table 6:** Univariate factor analysis of pathologic complete responses of breast cancer.

Parameters		*t* value	errors	Wald value	*P*	OR	95% CI
Age, year		−1.174	0.578	1.603	0.026	0.107	0.035−1.361
Before menopause		0.584	0.473	1.315	0.032	2.382	0.824−5.238
ER		−1.286	0.625	5.788	0.016	0.222	0.094−0.771
PR		−0.314	0.613	0.307	0.033	0.732	0.345−2.242
HER2		0.66	0.378	1.741	0.157	1.969	0.807−4.818
T classification	T1 vs. T2	−0.516	0.321	1.051	0.039	0.203	0.265−1.62
	T1 vs. T3	−0.682	0.465	1.218	0.031	0.376	0.124−1.523
N classification	N0 vs. N1	−0.372	0.213	0.896	0.042	0.618	0.153−2.16
	N0 vs. N2	−0.902	0.215	0.674	0.038	0.894	0.165−3.892
	N0 vs. N3	−1.658	0.327	0.472	0.030	1.321	0.125−2.942

**Table 7:** Multivariate factor analysis of pathologic complete responses of breast cancer.

Parameters		*t* value	errors	Wald value	*P*	OR	95% CI
ER		−1.381	0.553	5.727	0.018	0.211	0.076−0.759
PR		−0.372	0.520	0.284	0.492	0.718	0.264−2.237
HER2		0.597	0.373	1.699	0.141	1.872	0.756−4.743
T staging	T1 vs. T2	−0.481	0.236	0.938	0.042	0.253	0.133−1.642
	T1 vs. T3	−0.689	0.312	1.124	0.037	0.315	0.103−1.512
N staging	N0 vs. N1	−0.491	0.219	0.743	0.046	0.411	0.185−2.279
	N0 vs. N2	−0.943	0.316	0.918	0.032	0.327	0.164−3.008
	N0 vs. N3	−1.625	0.189	1.256	0.023	0.281	0.085−2.073

### Tolerability and Toxicity Assessment

After administering the standard dose of neoadjuvant treatment according to the NCCN guidelines, the dosage was adjusted in 53 patients because of adverse reaction to the drug, namely, grade four bone marrow suppression. This adverse reaction was significantly reduced after adjusting the dosage. The main toxicity occurred during chemotherapy (**Table 8**). PVL treatment did not increase the toxicity compared with that in CG (**Table 8**, *P* > 0.05). PVL treatment prevented side effects, such as neutrophil-reduced fever and anemia, caused by chemotherapy (**Table 8**, *P* > 0.05).

**Table 8:** Main side effects.

Parameters	EG	CG	*χ*^2^	*P* values
Grade 3/4, white blood cell reduction, *n* (%)	171(88.1)	165(91.2)	0.914	0.339
Grade 3/4, neutrophil reduction, *n* (%)	180(84.9)	173(81.6)	1.326	0.250
Grade 3, anemia, *n* (%)	3(1.4)	2(0.9)	0.006	0.938
Grade 3/4, platelet drop, *n* (%)	1(0.5)	1(0.5)	0.436	0.509
Neutrophil-reduced fever, *n* (%)	31(14.6)	29(13.7)	0	0.991
Grade 2/3, vomiting, *n* (%)	11(5.2)	9(4.2)	0.090	0.764
Musculoskeletal pain, numbness, *n* (%)	28(13.2)	29(13.7)	0.183	0.668
Cardiac events (precordial discomfort or UKG/EKG changes), *n* (%)	12(5.7)	15(7.1)	0.619	0.431
Mucosal response, *n* (%)	3(1.4)	6(2.8)	0.609	0.435

### Long-Term Follow-Up of Survival Outcome

**FIGURE 2:**
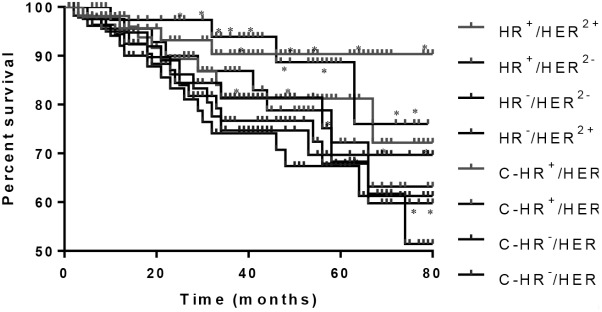
Mantel-Haenszel analysis of survival rate of breast cancer patients. HR^+^/HER2^−^, HR^−^/HER2^+^, HR^+^/HER2^+^, and HR^−^/HER2^−^ subtypes from EG group and C-HR^+^/HER2^−^, C-HR^−^/HER2^+^, C-HR^+^/HER2^+^, and C-HR^−^/HER2^−^ subtypes from CG group. ^∗^*P* < 0.05 vs. a control group.

The follow-up period of patients was ended on October 28, 2016, and the median follow-up time was 41 (8−112) months. The 3-year survival rate was 86.5% in patients from EG. In particular, the survival rates were 94.2% in HR^+^/HER2^−^ patients, 82.3% in HR^−^/HER2^+^ patients, 91.2% in HR^+^/HER2^+^ patients, and 75.9% in HR^−^/HER2^−^ patients (**Figure 2**). Meanwhile, the 3-year survival rate was 77.2% in patients from CG. In particular, the survival rates were 85.9% in HR^+^/HER2^−^ patients, 75.3% in HR^−^/HER2^+^ patients, 80.2% in HR^+^/HER2^+^ patients, and 73.1% in HR^−^/HER2^−^ patients (**Figure 2**). The results suggest that PVL may increase the survival rate of patients with BC.

## Discussion

High-performance liquid chromatography analysis showed that the main ingredients of PVL were caffeic and rosmarinic acids (**Figure 1B**). The results were consistent with previous reports: the main contents of PVL were rosmarinic acid and caffeic acid with high-level antioxidant capacities (Raafat et al., 2016; Qu et al., 2017; Chen et al., 2018). Caffeic acid exhibits anti-tumor and anti-inflammatory properties and was approved to inhibit the growth of BC (Chang et al., 2017; Kabala-Dzik et al., 2017). Rosmarinic acid also exhibits anti-inflammatory and anti-cancer properties, and anti-metastatic effect (Han et al., 2018). Thus, PVL improves the therapeutic outcomes of patients with BC possibly via its two main components.

After neoadjuvant chemotherapy, the clinical effective rate was 81.6%. The results of pathological evaluation showed that the total pCR rate was 22.1% among all cases, 51 cases (24.1%) from EG and 32 cases (15.1%) from CG (**Table 5**). Hence, all patients may benefit from neoadjuvant treatment, and PVL improves the therapeutic results of taxane.

In this experiment, the same chemotherapy protocol was administered among all patients to avoid other interfering factors on pCR rate between the two groups. Factors affecting pCR were also analyzed. Univariate analysis showed that ER and PR status was significantly correlated with patient’s pCR rate. Multivariate analysis showed that ER status was an independent factor of patient’s pCR rate. Taxane treatment may cause cardiotoxicity (Ky et al., 2014; Putt et al., 2015), which leads to some serious concerns about the safety of the medicine. In the present study, statistical analysis showed that the main dose-limiting toxicity for adverse reactions was hematologic toxicity, with grade 3/4 leucocytes and neutropenia. In patients with neutropenia, no serious adverse event was noted after symptomatic treatment. Severe thrombocytopenia and anemia were rarely observed.In terms of non-hematological toxicity, the incidence rates of adverse reactions, such as vomiting, cardiotoxicity, mucositis, and neurotoxicity, were relatively low. No serious adverse events occurred in both groups. Thus, the adverse reactions of combination therapy and chemotherapy could be tolerated as long as standardized clinical management will be used.

Patients with BC who achieved pCR after neoadjuvant therapy had higher survival rates than those who did not achieve pCR (Wang-Lopez et al., 2015a). Our results also suggest that patients who achieved pCR had better overall prognosis than those who did not achieve pCR. pCR is used as a surrogate marker for long-term survival of patients with BC (Wang-Lopez et al., 2015b; Biswas et al., 2017) and may indicate better prognosis.

This study also analyzed factors affecting patients’ long-term survival. The 3-year survival rates of patients with subtypes HR^+^/HER2^−^ and HR^+^/HER2^+^ was higher than that those of patients with HR^−^/HER2^+^ and HR^−^/HER2^−^ subtypes in EG (**Figure 2**). Similar results were also found in CG (**Figure 2**). Meanwhile, PVL treatment also increased the survival rate in EG compared with that in CG. All these results suggest that BC subtypes will affect the survival rate of patients. Moreover, PVL can improve the survival rate in all patients regardless of the subtype of BC.

This work presents evidence that pCR might be an alternative beneficial factor for improving the treatment outcomes of patients with BC. pCR could be a potential indicator for evaluating the efficacy of neoadjuvant treatment. PVL could increase the pCR and survival rates of patients with BC. For HER2-positive patients, the prognosis remains poor even after receiving standard anti-HER2 antibody therapy. In China, many patients are unable to receive anti-HER2 antibody therapy for economic reasons. Therefore, these patients are recommended to receive five to seven cycles of neoadjuvant therapy. We also recommend the use of anti-HER2 antibody therapy before surgery as the standard treatment for these BC patients (Kanojia et al., 2015). The results of this study support the use of PVL combined with taxane as the optimal therapy for neoadjuvant treatment of patients with BC. This regimen has significant efficacy and tolerable adverse reactions.

This work has several limitations: (1) Although neoadjuvant treatment has become a basic requirement for patients with BC, specific protocols for the treatment should be considered for individual patients. The current guidelines may be not recommended for some patients with BC. (2) The PVL oral solution contains two main ingredients, and the one that exerted the main function in the therapy remains unknown. (3) PVL also contains flavonoids, pentacyclic triterpenes, organic acids, polysaccharides, and volatile oils; however, only two ingredients were detected in the oral solution of PVL in this study. (4) PVL was not used for therapy of advanced BC. Further work is needed to address these issues in the future.

## Conclusion

Taxane and PVL chemotherapy showed high efficacy and safety for treatment of patients with BC. pCR was established as prognostic factor for patients with BC. Neoadjuvant therapy remains the standard intervention for patients with locally advanced and operable BC. Overall, the combination of oral administration of PVL and neoadjuvant therapy exhibits potential for BC therapy.

## Author Contributions

JZ and DJ designed the study. XZ and LZ performed the experiment. XL analyzed the data. XF wrote the manuscript. XL and XF revised and corrected the manuscript. All authors read and approved the final manuscript.

## Conflict of Interest Statement

The authors declare that the research was conducted in the absence of any commercial or financial relationships that could be construed as a potential conflict of interest.
